# Gut microbiota–immunity cascade in hepatocellular carcinoma: mechanisms and therapeutic opportunities

**DOI:** 10.3389/or.2025.1687901

**Published:** 2026-01-29

**Authors:** Jihao Yang, Yishuang Dai, Jia Li

**Affiliations:** 1 School of Acupuncture and Tuina, Guizhou University of Traditional Chinese Medicine, Guiyang, China; 2 Chongqing Medical and Pharmaceutical College, Chongqing, China

**Keywords:** gut microbiota, gut–liver axis, hepatocellular carcinoma, immune evasion, tumor immunemicroenvironment

## Abstract

Hepatocellular carcinoma (HCC) constitutes a major global health burden, with limited responsiveness to current immunotherapeutic regimens. Accumulating evidence underscores the gut microbiota as a crucial regulator of the gut–liver axis, modulating tumor initiation, immune evasion, and the outcomes of immunotherapeutic interventions—and notably, it concurrently exhibits both potential diagnostic biomarker value and actionable therapeutic target properties. In the present review, we synthesize the characteristic features of gut dysbiosis in HCC, delineate the mechanisms by which microbial metabolites—including short-chain fatty acids (SCFAs), bile acids, and indoles—modulate the tumor immune microenvironment (TME), and elaborate on their dual roles in promoting anti-tumor immunity while concomitantly mediating immune suppression. We further examine the clinical correlations between specific microbial taxa and the efficacy of immune checkpoint inhibitors (ICIs)—findings that support the utility of gut microbiota signatures as predictive or diagnostic biomarkers—and explore emerging microbiota-targeted strategies, such as fecal microbiota transplantation (FMT), probiotic supplementation, phage therapy, and dietary modulation, which validate the gut microbiota as a viable therapeutic target.

## 1 Introduction

HCC, the predominant subtype of primary liver cancer (accounting for 75%–85% of all cases), ranks as the sixth most prevalent malignancy and the third leading cause of cancer-related mortality worldwide. Given its high incidence and mortality, HCC imposes a substantial public health burden globally (1). The initiation and progression of HCC are driven by intricate interactions across multiple factors, encompassing persistent inflammatory injury, hepatic fibrosis, dysregulation of oncogenes and tumor suppressor genes (2, 3), perturbations in the immune microenvironment (4, 5), metabolic reprogramming (6), and the accumulation of genetic and epigenetic alterations. Collectively, these processes contribute to the marked molecular heterogeneity and clinical complexity that characterize HCC (7).

Currently, receptor tyrosine kinase inhibitors such as sorafenib and lenvatinib remain the standard first-line therapeutic options for advanced HCC (8). More recently, the combination of the anti–PD-L1 antibody atezolizumab and the anti–VEGF antibody bevacizumab has demonstrated significant superiority over sorafenib in terms of overall survival (OS), progression-free survival (PFS), and objective response rate (ORR) (9, 10). The HIMALAYA trial further revealed that dual immunotherapy with durvalumab (an anti–PD-L1 agent) and tremelimumab (an anti–CTLA-4 agent) achieved a 4-year OS rate of 25.2%, thereby expanding the available treatment armamentarium for HCC (11). Nevertheless, the prognosis of HCC remains dismal: only approximately 30% of patients achieve objective responses to current therapies, the 3-year OS rate remains below 50% (12), and response rates to monotherapy with ICIs are as low as 15%–20% (13). The challenges of immunotherapy resistance, low response rates, limited predictive biomarkers, and insufficient understanding of the relationship between the TME and treatment efficacy underscore the urgent need to dissect the immune regulatory networks governing HCC.

In recent years, a growing body of evidence has identified the gut microbiota—a central regulator of the “gut–liver axis—as a key mediator of HCC pathogenesis. Beyond contributing to tumor initiation and progression via microbial metabolites, the gut microbiota also modulates therapeutic responses through immune regulation. However, the cascade mechanisms linking the microbiota, its metabolites, and immunotherapeutic responses have not been systematically integrated. To address this knowledge gap, the present review provides the first comprehensive synthesis of the “microbiota–metabolite–immunotherapy” cascade in HCC. We systematically examine the molecular mechanisms through which the gut microbiota regulates immune homeostasis via the gut–liver axis, analyze its roles in facilitating immune evasion and shaping responsiveness to immunotherapy, and discuss strategies for microbiota-targeted modulation to optimize HCC immunotherapy. Our overarching aim is to establish a theoretical framework that can overcome current clinical bottlenecks in HCC treatment.

## 2 Physiological basis and pathological associations of the gut microbiota and gut-liver axis

### 2.1 Physiological characteristics of the gut microbiota and functional framework of the gut-liver axis

**FIGURE 1:**
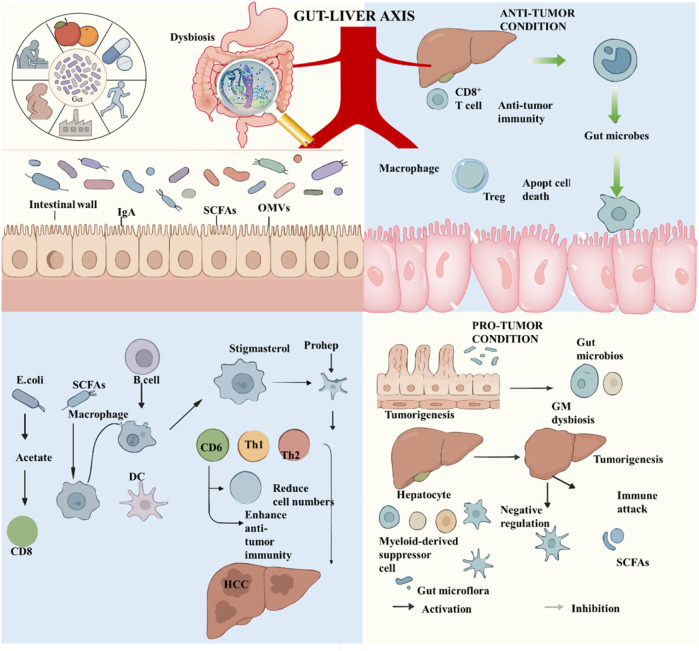
Regulatory network of the Gut-Liver Axis in anti-tumor and pro-tumor microenvironments. Upper Left: Triggers & Barrier Function. Factors like diet and medication can cause gut microbiota dysbiosis. The intestinal barrier contributes to gut-liver axis signaling by secreting IgA, SCFAs, and OMVs. Upper Right: Anti-Tumor Immunity. Via the gut-liver axis, the microbiota activates hepatic immunity, promoting CD8^+^ T cell and macrophage-mediated tumor killing. Tregs maintain immune balance for a coordinated anti-tumor response. Lower Left: Anti-HCC Pathways. SCFAs (e.g., acetate from *E. coli*) modulate immune cells like macrophages and DCs, shifting the balance toward pro-inflammatory Th1 responses. Stigmasterol also enhances anti-HCC immunity by binding to CD6^+^ cells. Lower Right: Pro-Tumor Mechanisms. Gut dysbiosis initiates intestinal tumorigenesis and subsequently drives HCC progression via the gut-liver axis. An immunosuppressive microenvironment emerges, characterized by MDSC accumulation and SCFAs that inhibit T cell activity, creating a pro-tumor cycle.

The human gastrointestinal tract harbors approximately 100 trillion microorganisms, forming a highly complex microecosystem. Among these microbes, *Firmicutes* and *Bacteroidetes* are the dominant phyla (collectively accounting for >90% of the total microbial community), supplemented by other phyla such as *Actinobacteria* and *Proteobacteria* (14). These microorganisms establish symbiotic relationships with the host through metabolic cooperation, immune education, and maintenance of intestinal barrier integrity, thereby regulating nutrient absorption, metabolic balance, and mucosal immune homeostasis (Figure 1).

The gut-liver axis functions as a bidirectional communication system that links the intestine and the liver, relying on three core mechanisms: (1) The portal venous system, which drains approximately 70% of intestinal blood, enabling microbial products and metabolites to reach the liver directly (15, 16); (2) Bile acids secreted by the liver, which enter the intestine via the biliary tract, undergo microbial metabolism, and subsequently re-enter the liver to regulate bile acid homeostasis (17); (3) Neuro-immune signaling mediated by the vagus nerve, lymphatic vessels, and cytokines (18). Under physiological conditions, the intestinal barrier (comprising tight junctions and mucosal lymphocytes) and hepatic immune defenses (including Kupffer cells and liver sinusoidal endothelial cells) synergistically prevent microbial translocation, thereby maintaining the homeostasis of the gut-liver axis (19). Once this balance is disrupted—for instance, by dysbiosis or increased intestinal permeability—pathological cascades are triggered, driving the initiation and progression of HCC (20, 21).

### 2.2 Characteristics of gut microbiota dysbiosis in HCC patients

In HCC, the balance of the gut microbiota and the functionality of the gut-liver axis are profoundly disrupted, leading to distinct patterns of dysbiosis. β-diversity analysis reveals a significant separation between the microbial communities of HCC patients and healthy individuals (*R* ^2^ = 0.152, P = 0.001), indicating a systemic remodeling of the microbial community structure (22). This shift not only involves changes in the abundance of specific taxa but also reflects disruptions in microbial interaction networks—including the breakdown of cooperative relationships between dominant and commensal bacteria—thereby impairing the regulatory capacity of the gut-liver axis.

At the phylum level, HCC patients typically exhibit increased abundance of the pro-inflammatory phylum *Proteobacteria*, whereas the relative abundances of *Firmicutes*, *Verrucomicrobia*, and certain subtypes of *Actinobacteria* are significantly reduced (23, 24). Dysbiosis patterns also vary according to disease stage and etiology: patients with advanced HCC display enrichment of *Proteobacteria* and *Patescibacteria*, accompanied by progressive loss of *Actinobacteria* (23); patients with HBV-related HCC show reduced levels of Proteobacteria, while those with HCV-related HCC (derived from HCV-associated cirrhosis) exhibit elevated Proteobacteria abundance, suggesting that etiological factors shape microbial dysbiosis patterns during cirrhosis-to-HCC progression and thereby influence HCC development through distinct microbial pathways (25).

At the genus level, dysbiosis exhibits greater specificity: pathogenic genera such as *Escherichia/Shigella*, *Enterobacter*, *Klebsiella*, and *Haemophilus* are significantly enriched, whereas probiotic genera with immunomodulatory or metabolic protective roles—including *Lactobacillus*, *Bifidobacterium*, *Bacteroides*, and the Lachnospiraceae NK4A136 group—are depleted (26, 27). Even in early-stage HCC, clear differences in microbial composition are observed: 13 genera (including *Gemmiger* and *Parabacteroides*) are increased, while 12 genera (including *Alistipes* and *Phascolarctobacterium*) are decreased (24). Critically, bacteria that produce pro-inflammatory lipopolysaccharide (LPS) are enriched, whereas those that generate anti-inflammatory metabolites (e.g., butyrate) are reduced, directly fostering a local pro-carcinogenic microenvironment within the liver (28).

### 2.3 Core mechanisms by which the gut microbiota regulates HCC through the gut-liver axis

The central mechanism by which dysbiosis promotes HCC via the gut-liver axis can be summarized as a cascade of “barrier disruption → microbial translocation → hepatic pathological remodeling.” This cascade involves intestinal barrier damage, chronic inflammation, metabolic imbalance, immune suppression, and epigenetic interactions between the host and microbiota:

Intestinal barrier damage serves as the initiating step. Factors such as a high-fat diet, alcohol consumption, and viral infection can disrupt intestinal tight junctions (e.g., by decreasing the expression of Occludin and Claudin), thereby increasing intestinal permeability (29, 30). Dysbiosis—for example, the expansion of *Proteobacteria*—further exacerbates barrier injury by producing metabolites such as LPS, enabling microbial products (including LPS and lipoteichoic acid) to translocate to the liver via the portal vein (28, 31). These ectopic microbial signals continuously stimulate hepatic innate immune cells, initiating the developmental cascade of HCC.

Chronic inflammation arises from the recognition of microbial components by hepatic immune cells. LPS activates the TLR4/NF-κB pathway, upregulating the expression of proto-oncogenes such as C-Myc and Cyclin D1 to induce DNA damage and mutations in hepatocytes; lipoteichoic acid activates toll-like receptors (TLRs) in hepatic stellate cells, promoting the secretion of IL-6 and IL-17 and accelerating tumor cell proliferation via the STAT3/Cyclin E signaling axis (32); the enrichment of pro-inflammatory Enterobacteriaceae strengthens the hepatic pro-inflammatory milieu, facilitating the transformation of cirrhosis to HCC (33).

Metabolite dysregulation reshapes hepatic pathology. Reduced SCFA levels impair intestinal barrier integrity and weaken anti-inflammatory signaling (26, 34, 35). Secondary bile acids (e.g., DCA) disrupt lipid metabolism via FXR/TGR5 signaling, while dysbiosis (e.g., the overgrowth of *E. coli*) causes abnormal bile acid profiles, promoting hepatic steatosis and carcinogenesis (17). Additionally, LPS promotes angiogenesis and tumor cell proliferation by activating inflammatory pathways (28).

Epigenetic regulation links microbial metabolites to the expression of host genes. Butyrate enhances the transcription of the tumor suppressor gene *TP53* by inhibiting histone deacetylase (HDAC) activity (34), whereas DCA activates β-catenin (encoded by *CTNNB1*), promoting the expression of oncogenes (17). Immune suppression is further reinforced by dysbiosis: pathogenic metabolites (e.g., those derived from *Klebsiella*) inhibit the infiltration and activity of *CD8* ^*+*^ T cells while expanding Treg populations, creating an immunosuppressive TME (36). Intratumoral microbiota such as *Ruminococcus* further accelerate HCC progression by inducing the release of TNF-α (37).

## 3 Interaction mechanisms between the gut microbiota and the HCC immune microenvironment

**FIGURE 2:**
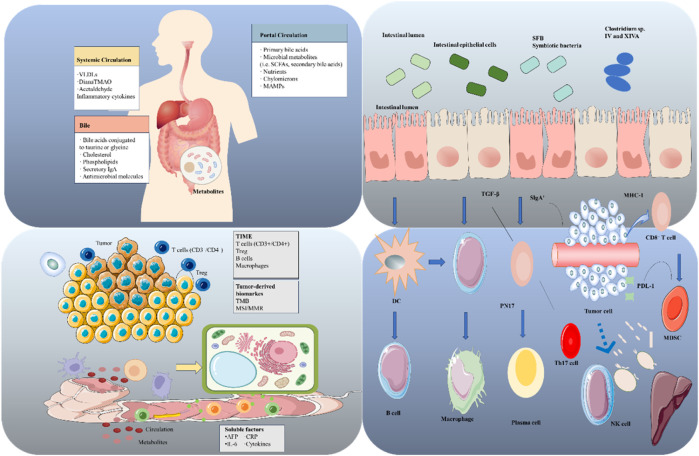
Cross-organ interaction network of “gut microbiota → metabolic circulation → tumor microenvironment (TME) → immune regulation.” Upper left quadrant: Molecular bridges of metabolic circulation. The systemic circulation transports TLR ligands (e.g., LPS), SCFAs, and cytokines, mediating distant immune regulation. The portal circulation delivers bile acids, nutrients, and microbe-associated molecular patterns (MAMPs), directly linking the gut and liver. Bile acids form a feedback loop consisting of “bile → microbiota → metabolites → liver/systemic circulation.” Upper right quadrant: Intestinal “immune sentinels.” Commensal bacteria interact with intestinal epithelial cells; regulatory signals (including TGF-β, SIgA, and MHC-I antigen presentation) determine local immune balance, indirectly shaping tumor immunity. Lower left quadrant: Tumor battlefield ecology. The TME comprises tumor cells, immune cells, and tumor-derived molecules. Circulating metabolites may exert pro-carcinogenic effects (e.g., aflatoxin, CRP) or anti-tumor effects (e.g., vitamin D, polyphenols). Lower right quadrant: Immune battle processes. DCs activate B cells, macrophages, neutrophils, and Th17 cells to initiate immune responses. Tumor evasion strategies include the expression of PD-L1, the infiltration of MDSCs, and the dysfunction of NK cells. Microbial metabolites such as SCFAs regulate DC maturation and Th17 differentiation, tipping the balance between immune activation and evasion.

The TME of HCC is characterized by profound immunosuppression, marked by the infiltration of large numbers of suppressive immune cells (e.g., tumor-associated macrophages [TAMs], regulatory T cells [Tregs]) and the presence of inhibitory signaling molecules. Together, these elements form an immunosuppressive barrier that facilitates tumor immune evasion (38). Through the gut-liver axis, the gut microbiota regulates the differentiation and function of diverse immune cell populations within the TME, establishing a complex “microbiota–immunity–tumor” interaction network (Figure 2).

### 3.1 Targeted regulation of HCC immune cells by the gut microbiota

#### 3.1.1 Functional regulation of T Cells and natural killer (NK) cells

*CD8* ^*+*^ cytotoxic T cells, the central effector cells of anti-tumor immunity, are profoundly influenced by the gut microbiota, which modulates their activity and functional status to directly affect tumor clearance. For example, acetate secreted by *Bacteroides thetaiotaomicron* enhances the cytotoxicity of *CD8* ^*+*^ T cells and their secretion of IFN-γ, thereby promoting the killing of tumor cells (39). Similarly, 2,5-dimethylcelecoxib enriches the abundance of *Acidaminococcus* and reduces PD-1 expression in *CD8* ^*+*^ T cells via the AMPK/mTOR pathway, effectively reversing T cell exhaustion and restoring anti-tumor function (40).

For *CD4* ^*+*^ T cell subsets, the microbiota influences immune balance by regulating their differentiation. *Limosilactobacillus reuteri* activates Th1 immune responses via its metabolite indole-3-aldehyde, thereby promoting anti-tumor immunity, while concurrently inhibiting Th2-mediated pro-tumor inflammation (41).

Innate immunity is likewise regulated by the gut microbiota: *Bacteroides uniformis* and *Bifidobacterium bifidum* restore the cytotoxicity of NK cells by enhancing the expression of granzyme B (42), while *Lactobacillus plantarum* increases the secretion of IL-22 in NK cells, augmenting tumor cell clearance and complementing adaptive immune responses (43).

#### 3.1.2 Regulation of macrophage polarization balance

Macrophage polarization is a key determinant of the TME phenotype. M1 macrophages exert anti-tumor effects through the secretion of TNF-α and IL-1β, whereas M2 macrophages promote angiogenesis and release suppressive cytokines, thereby accelerating tumor progression (44–46). The gut microbiota can direct macrophage polarization via its metabolites: D-lactic acid produced by *Lactobacillus delbrueckii* promotes the repolarization of M2 macrophages to an M1 phenotype via the TLR2/TLR9–PI3K/Akt pathway, enhancing anti-tumor immunity (13); conversely, SCFAs may recruit M2 macrophages through GPR43 signaling, promoting the formation of an immunosuppressive TME (47). This bidirectional regulation highlights the fine-tuning capacity of the gut microbiota in shaping macrophage function and the immune phenotype of the TME.

#### 3.1.3 Functional remodeling of antigen-presenting cells

Dendritic cells (DCs)—the bridge between innate and adaptive immunity—rely on their maturation status to initiate effective T cell responses, a process that is strongly influenced by the gut microbiota. *Bifidobacterium* spp. enhance the antigen-presenting capacity of DCs by upregulating the expression of CD80/CD86, thereby driving the infiltration of *CD8* ^*+*^ T cells into tumors (13). Similarly, the probiotic *Escherichia coli* Nissle 1917 induces DCs to secrete IL-12, amplifying Th1 immune responses and providing priming signals for adaptive immunity (48). Thus, the modulation of DC function represents a pivotal node linking gut ecology to systemic anti-tumor immunity. Specifically, microbiota-activated mature DCs not only prime CD8^+^ T cell differentiation into cytotoxic T lymphocytes via IL-12 secretion but also secrete TNF-α to promote M1 macrophage polarization, forming a synergistic anti-tumor immune loop (13, 48).

#### 3.1.4 Abnormal activation of immunosuppressive cells

Myeloid-derived suppressor cells (MDSCs) and Tregs—major immunosuppressive cell types within the TME—play central roles in HCC immune evasion, and their activation is strongly influenced by the gut microbiota. Dysbiosis promotes the hepatic recruitment of MDSCs via the TLR4/NF-κB pathway, establishing a vicious “inflammation–immunosuppression” cycle with IL-6 and IL-1β that progressively diminishes anti-tumor immunity (49–51). The SCFA butyrate strengthens Treg function via histone acetylation at the *Foxp3* locus (52, 53), while outer membrane vesicles derived from *Bacteroides fragilis* induce Tregs to secrete IL-10, consolidating the immunosuppressive microenvironment (54, 55).

### 3.2 Integrated effects of the inflammation–immunity synergistic network

The interaction between inflammation and immunity constitutes the core mechanism by which the gut microbiota modulates the HCC TME. This interaction is manifested in the crosstalk of signaling pathways, the regulation of metabolic networks, and the metabolic reprogramming of immune cells.

On one hand, microbial components such as LPS and peptidoglycan activate downstream signaling pathways through TLRs. For example, the LPS–TLR4 axis stimulates Kupffer cells to produce TNF-α and IL-6, while simultaneously inducing the expression of PD-L1, which suppresses T cell function (56, 57). Conversely, the activation of TLR5 promotes DC maturation via MyD88, partially counteracting immunosuppression (58). This dual regulation underscores the complexity of microbiota–immune interactions in the context of HCC.

On the other hand, gut microbes influence immune metabolism. *Akkermansia muciniphila* exemplifies a bacterium with dual regulatory effects: it reduces liver inflammation by repairing the intestinal barrier (thereby decreasing LPS translocation) while simultaneously enhancing anti-tumor immunity through IL-12–dependent crosstalk between DCs and T cells (59).

Microbial metabolites also remodel the metabolism of immune cells. Acetate derived from *Bacteroides thetaiotaomicron* reverses the Warburg effect in *CD8* ^*+*^ T cells by inhibiting glycolytic enzymes (e.g., hexokinase), enhancing mitochondrial oxidative phosphorylation and T cell cytotoxicity (39). In contrast, lactic acid produced by *Enterococcus* activates the mTOR–HIF1α pathway in Tregs, reinforcing their glucose-dependent metabolism and immunosuppressive function (55). Additionally, microbial metabolites can activate the cyclic GMP–AMP synthase (cGAS)/stimulator of interferon genes (STING) pathway, which in turn reshapes type I interferon signaling within the TME (48).

## 4 Mechanisms by which the gut microbiota influences HCC through regulation of immune evasion

Immune evasion is a defining hallmark of HCC, whereby tumor cells reshape the TME and alter their own phenotypes to escape immune surveillance. This process is a key driver of malignant progression and therapeutic resistance. Given the extensive regulatory effects of the gut microbiota on the HCC immune microenvironment, the gut microbiota—via the gut-liver axis—further contributes to immune evasion through multiple pathways, thereby serving as a critical bridge between intestinal dysbiosis and hepatic immune dysfunction (60–62).

### 4.1 Core mechanisms of HCC immune evasion: synergistic effects of the microenvironment and tumor cells

Immune evasion in HCC is a multi-faceted process driven by the dynamic interaction between the TME and tumor cells themselves. To understand how the gut microbiota regulates this process, it is first necessary to clarify the core endogenous mechanisms of HCC immune evasion, which provide the biological context for the subsequent regulatory role of the gut microbiota. Immune evasion in HCC is not mediated by a single factor but arises from dynamic interactions between the TME and the intrinsic properties of tumor cells. Central mechanisms include the formation of an immunosuppressive TME, the role of tumor-initiating cells (TICs), the intrinsic immune-evasion phenotypes of tumor cells, and microbiota-driven immune editing—all of which collectively establish an “immune shelter” for tumor cells.

#### 4.1.1 Construction of an immunosuppressive TME

The intrinsic immune tolerance of the liver provides a baseline permissive environment for tumor immune escape. In HCC, this tolerance is reinforced by a suppressive cellular network within the TME. Kupffer cells, Tregs, and MDSCs form a fundamental suppressive axis: TAMs inhibit the cytotoxicity of CD8^+^ T cells by secreting IL-10 and TGF-β and by upregulating PD-L1 expression. In parallel, neutrophils release arginase 1, depleting arginine and thereby impairing T cell proliferation (31, 63, 64). These suppressive cells cooperate with extracellular matrix components (e.g., collagen, hyaluronic acid) to establish both physical and functional barriers that block anti-tumor immune responses.

#### 4.1.2 Immune evasion driven by tumor-initiating cells

On the basis of the immunosuppressive TME, tumor-initiating cells (TICs)—a subpopulation of tumor cells with potent tumorigenic and drug-resistant properties—act as key engines of immune evasion, further exacerbating the escape of tumor cells from immune surveillance (65, 66). CD49f^+^ TICs recruit pro-tumor neutrophils via the CXCL2–CXCR2 axis, forming a positive feedback loop in which neutrophil-derived CCL4 reprograms surrounding tumor cells into a TIC phenotype (67, 68). TICs further evade recognition and killing by CD8^+^ T cells by upregulating the expression of CD155 (69, 70). Additionally, activation of the WNT–β-catenin pathway reduces the expression of antigen presentation molecules, lowering the immunogenicity of TICs and facilitating immune escape.

#### 4.1.3 Intrinsic immune evasion characteristics of tumor cells

Beyond the influence of the TME and TICs, genetic and epigenetic alterations in tumor cells themselves confer inherent immune-evasive traits, which are an important intrinsic factor in HCC immune evasion. Mutations in TP53 diminish the sensitivity of tumor cells to cytotoxic T cells by inhibiting IFN-γ signaling, while activation of β-catenin suppresses the secretion of CXCL10, reducing the infiltration of T cells into the TME (71). Moreover, tumor cells upregulate the expression of checkpoint molecules such as PD-L1 and B7-H3, which function as molecular shields to block T cell recognition and activation, thereby establishing autonomous immune evasion mechanisms (72, 73).

#### 4.1.4 Role of microbiota in immune editing

The gut microbiota is not only a regulator of the above endogenous immune evasion mechanisms but also participates in the immune editing process of tumors, shaping the clonal evolution of HCC cells and thereby influencing their immune evasion potential. The gut microbiota contributes to immune editing by shaping the clonal evolution of tumors. Initially, microbial metabolites (e.g., LPS) enhance immune surveillance via the activation of TLR4, eliminating highly immunogenic tumor clones. However, prolonged dysbiosis—for example, the depletion of butyrate-producing bacteria—weakens immune clearance by reducing anti-tumor cytokine secretion (e.g., IFN-γ), allowing poorly immunogenic clones to dominate (36). For instance, the enrichment of *Klebsiella pneumoniae* can induce epigenetic silencing of HLA-A in tumor cells, reducing their recognition by CD8^+^ T cells and enabling these clones to prevail after immune selection (36).

### 4.2 Key pathways by which the gut microbiota regulates HCC immune evasion

Having clarified the core endogenous mechanisms of HCC immune evasion, it is evident that the gut microbiota intervenes in this process through multiple key pathways via the gut-liver axis. These pathways connect intestinal dysbiosis to hepatic immune dysfunction, directly driving the immune evasion of HCC cells.

#### 4.2.1 Intestinal barrier damage and activation of immunosuppressive signals

The integrity of the intestinal barrier is the first line of defense against microbial translocation, and its damage is the initial step by which the gut microbiota initiates the regulation of HCC immune evasion. The integrity of the intestinal barrier relies on the synergy between the microbiota and mucosal immunity. Dysbiosis—particularly the expansion of Proteobacteria—disrupts the expression of tight junction proteins (e.g., Occludin, ZO-1), increasing intestinal permeability (69, 70),a phenomenon also observed in cirrhotic portal hypertension (CPH, a key HCC precursor) where gut microbiota dysbiosis similarly impairs intestinal barrier function (74). Consequently, microbial products such as LPS and peptidoglycan translocate to the liver via the portal vein. Chronic stimulation of hepatic immune cells and tumor cells via TLRs induces “LPS tolerance,” which involves the activation of the SMAD/STAT3 pathway and the secretion of factors such as TGF-β and hepatocyte growth factor (HGF). These factors inhibit DC maturation and T cell activity, thereby fostering an immunosuppressive TME (75, 76).

#### 4.2.2 Differentiation and functional enhancement of immunosuppressive cells

Intestinal barrier damage and the subsequent activation of immunosuppressive signals further drive the differentiation and functional enhancement of immunosuppressive cells in the liver, which is another key pathway by which the gut microbiota promotes HCC immune evasion, forming a “barrier damage–signal activation–suppressive cell expansion” cascade. Gut microbes promote the expansion and activation of suppressive cell populations, reinforcing immune evasion. Certain pathogenic bacteria (e.g., *Enterococcus*) induce the recruitment of IL-10^+^ Tregs in the liver, thereby suppressing the clonal expansion of CD8^+^ T cells (77). Secondary bile acids such as isoalloLCA enhance the expression of FOXP3 via mitochondrial ROS signaling, while isoDCA indirectly augments Treg function through the activation of vitamin D receptor (VDR) in DCs (75, 78).

Similarly, gut dysbiosis drives the accumulation of MDSCs in the liver via TLR4/NF-κB signaling (79). MDSCs suppress T cell proliferation by producing reactive oxygen species (ROS) and inducible nitric oxide synthase (iNOS) and establish a feedback loop with IL-6 that perpetuates inflammation and immunosuppression (49–51). Studies have shown that IL-6 exerts its effects in the liver through its pro-inflammatory functions, promoting liver fibrosis, steatosis, and carcinogenesis, indicating that IL-6 is one of the key drivers of hepatocellular carcinoma development (80). High serum IL-6 levels are a key indicator of inflammatory dysregulation, and a state of persistent elevation is directly associated with an increased risk of hepatocellular carcinoma progression (81). This mechanism synergizes with TIC-mediated neutrophil recruitment, collectively destabilizing immune homeostasis in the liver.

#### 4.2.3 Regulation of immune checkpoints and tumor cell phenotypes

In addition to regulating the intestinal barrier and immunosuppressive cells, the gut microbiota and its metabolites also directly modulate the expression of immune checkpoints and the phenotypic characteristics of tumor cells, which is a more direct pathway to promote HCC immune evasion, linking microbial regulation to the intrinsic immune evasion traits of tumor cells. The gut microbiota and its metabolites enhance tumor immune evasion by regulating the expression of immune checkpoints and modifying tumor cell phenotypes. Dysbiosis activates the EGFR/RAS pathway, driving the overexpression of PD-L1 on tumor cells (82, 83). Meanwhile, reduced SCFA levels—resulting from the depletion of butyrate-producing bacteria—diminish the epigenetic repression of PD-L1 (via reduced HDAC inhibition), further elevating PD-L1 expression (84). Similarly, microbial metabolites such as LPS stabilize the expression of CD155 in TICs via the activation of STAT3, maintaining the resistance of TICs to killing by CD8^+^ T cells (36).

### 4.3 Dual roles of microbiota metabolites in immune evasion

The above pathways by which the gut microbiota regulates HCC immune evasion are largely mediated by its metabolites, which are the key molecular messengers of the gut-liver axis. Notably, these microbial metabolites do not have a unidirectional regulatory effect on immune evasion but instead exhibit dual roles, with their balance determining the ultimate trend of tumor immune escape. Microbial metabolites function as bidirectional regulators of immune evasion: LPS chronically activates TLR4/NF-κB signaling, inducing the secretion of VEGF and IL-8 to promote angiogenesis and recruit suppressive cells (24). Secondary bile acids such as deoxycholic acid (DCA) drive the polarization of M2 macrophages, reinforcing immunosuppression through the secretion of IL-10 (85, 86); in contrast, butyrate enhances the antigen presentation capacity of tumor cells (e.g., by upregulating MHC-I expression) via HDAC inhibition (87). Isobutyric acid, produced by Bifidobacterium, induces the secretion of IFN-γ by CD8^+^ T cells, thereby reversing T cell exhaustion (88). Thus, the balance between pro-evasion and anti-evasion metabolites—tightly regulated by the composition of the gut microbiota—critically determines the extent of immune escape in HCC.

## 5 Mechanisms by which the gut microbiota regulates HCC through influencing immunotherapy response

The introduction of ICIs has transformed the treatment landscape for advanced HCC. However, limited clinical response rates (15%–20% with monotherapy and approximately 30% with combination therapy) and the emergence of therapeutic resistance remain major obstacles (89–92). Given the central role of the gut microbiota in modulating immune evasion, alterations in microbial composition and function are closely associated with immunotherapeutic outcomes, positioning the microbiota as a potential target to enhance therapeutic efficacy.

### 5.1 Clinical associations between gut microbiota characteristics and HCC immunotherapy response

Clinical studies demonstrate that the diversity of the gut microbiota and the relative abundance of specific genera can serve as predictive biomarkers for the prognosis of immunotherapy. Notably, treatment outcomes are linked not only to the baseline microbial composition but also to dynamic changes in the microbiota during therapy. For example, in patients receiving anti–PD-1 therapy, responders exhibit significant differences in microbial diversity before and after treatment (P = 0.0274), whereas baseline α-diversity does not differ significantly between responders and non-responders (93). β-diversity analyses reveal a clear separation in microbial community structure between responders and non-responders, indicating that systemic shifts in the microbiota may underlie the heterogeneity of therapeutic responses.

Reduced microbial diversity is frequently accompanied by the enrichment of immunosuppressive species (e.g., *K. pneumoniae*), whereas higher diversity supports a more balanced anti-tumor immune response (31) (Table 1).

**TABLE 1:** Clinical trials and innovative therapies for HCC.

Name	Type	Modality	Target	Phase
EMERALD-1 (NCT03778957)	Clinical trial	Durvalumab + bevacizumab + TACE vs. TACE ± Placebo	PD-L1, VEGF	Phase Ⅲ
LEAP-012 (NCT04246177)	Clinical trial	Pembrolizumab + lenvatinib + TACE vs. TACE ± Placebo	PD-1, VEGFRs	Phase Ⅲ
SCG101 (NCT06617000)	Cell therapy trial	HBV-specific TCR-T	HBV-related antigens	Phase I
AZD-7003 (NCT05155189)	Cell therapy trial	GPC3-targeted CAR-T	GPC3	Phase I
CT011 (NCT02395250)	Cell therapy trial	GPC3-targeted CAR-T	GPC3	Phase I
MG4101 (NCT02008929)	Cell therapy trial	Allogeneic NK cells	Innate immunity	Phase II
Domvanalimab	Drug development	Checkpoint inhibitor (TIGIT inhibitor)	TIGIT	Preclinical
Anti-TIM3 antibody	Drug development	Checkpoint inhibitor (TIM-3 inhibitor)	TIM-3	Preclinical
GS-0151	Drug development	Anti-PD-1 antibody	PD-1	Phase I
CD147-ADC	Drug development	ADC	CD147	Pre-IND
Dual-target CAR-T	Cell therapy development	CAR-T (GPC3/AFP dual-targeted)	GPC3/AFP	IND-enabling

Several genera exhibit quantitative associations with therapeutic efficacy: the presence of *Akkermansia muciniphila* correlates with improved survival in patients receiving anti–PD-1 treatment (hazard ratio [HR] = 0.32, 95% confidence interval [CI]: 0.15–0.68) (31); an increased *Prevotella/Bacteroidetes* ratio and a reduced *Firmicutes/Bacteroidetes* ratio are associated with higher ORR and prolonged PFS; a high abundance of Lachnospiraceae and a low abundance of Prevotellaceae predict longer PFS, whereas the enrichment of Veillonellaceae predicts a poor prognosis (HR = 2.1, 95% CI: 1.2–3.6) (94, 95). Additionally, *Alistipes* sp. Marseille-P5997 and Lachnospiraceae bacterium GAM79 are enriched in responders, correlating with extended OS (96).

In addition, several ongoing clinical trials are explicitly investigating the role of gut microbiota in the treatment of hepatocellular carcinoma, such as NCT04729322 (FMT combined with nivolumab in advanced solid tumors), NCT05295962 (reversing drug resistance in unresectable hepatocellular carcinoma through FMT), and NCT05750030 (the FAB-HCC trial, which combines FMT with atezolizumab/bevacizumab for hepatocellular carcinoma with resistance to immunotherapy). These trials aim to verify the effectiveness of microbiota modulation as an adjunct to standard treatments.

### 5.2 Molecular mechanisms by which the gut microbiota influences HCC immunotherapy response

The gut microbiota influences ICI efficacy through multiple interconnected mechanisms, as elaborated below. First, in terms of immune cell recruitment and activation, Akkermansia muciniphila secretes metabolites that recruit CCR9^+^CXCR3^+^CD4^+^ T cells into the TME via IL-12–dependent mechanisms, while simultaneously increasing the infiltration of CD8^+^ T cells and the apoptosis of tumor cells (55); similarly, Limosilactobacillus reuteri colonizes tumors and produces indole-3-aldehyde (I3A), which activates the aryl hydrocarbon receptor (AhR) pathway in CD8^+^ T cells to promote the secretion of IFN-γ and enhance responses to ICIs (97), and Bifidobacterium enhances DC maturation by upregulating the expression of CD80/CD86, thereby stimulating the activation of CD8^+^ T cells and restoring the efficacy of PD-L1 inhibitors (13).

Second, metabolite-driven synergy plays a pivotal role: acetate promotes the polarization of M1 macrophages through ACC1-dependent fatty acid synthesis, indirectly enhancing the activity of CD8^+^ T cells (98); butyrate boosts the cytotoxicity and memory phenotypes of CD8^+^ T cells via epigenetic regulation (e.g., histone modification), thereby sustaining long-term immune responses (84); secondary bile acids upregulate the number of CXCR6^+^ NKT cells through enterohepatic circulation to strengthen innate immune surveillance (99); and inosine produced by *Lactobacillus* johnsonii activates Th1 cells via A2AR signaling, synergizing with immune checkpoint blockade (100).

Antigen cross-reactivity constitutes another critical mechanism: microbial antigenic peptides may cross-react with tumor antigens to activate broad-spectrum T cell responses, and tumor-infiltrating CD4^+^ T cell clones can recognize both tumor and microbial antigens, producing IFN-γ, GM-CSF, TNF, and granzyme B—factors that synergize with ICIs to enhance tumor clearance (101). Finally, microbial translocation further amplifies ICI efficacy: certain gut microbes translocate into tumors or peripheral organs to amplify local immune activation; for instance, A. muciniphila increases its systemic and intratumoral abundance, which augments anti-tumor responses and improves ICI efficacy (101–103).

### 5.3 Gut microbiota and HCC immunotherapy resistance

Gut dysbiosis is a critical driver of immunotherapy resistance, mediated by both the direct effects of pathogenic bacteria and the disruption of immune activation. On one hand, pathogenic pro-resistance mechanisms are supported by clinical evidence: *Pseudomonas* spp. and *Klebsiella pneumoniae* are enriched in non-responders to immunotherapy, and these bacteria secrete metabolites (e.g., specific lipids, signaling molecules) that inhibit T cell proliferation and cytotoxicity, thereby reducing ICI efficacy (104). Furthermore, heparin-binding EGF (HB-EGF) produced by certain bacteria (e.g., *E. coli*) activates the EGFR/RAS signaling pathway, driving the persistent overexpression of PD-L1 on tumor cells and enabling them to evade anti–PD-L1 therapy (55, 70).

On the other hand, dysbiosis disrupts immune activation: antibiotic-induced dysbiosis impairs cytotoxic T lymphocyte (CTL) responses; for instance, broad-spectrum antibiotics reduce the colonization of *Bacteroides* fragilis—a key inducer of CTL activity—leading to resistance to CTLA-4 blockade (54). Furthermore, dysbiosis alters the composition of immune cell populations, promoting the infiltration of regulatory T cells (Tregs) and MDSCs; these cells secrete IL-10 and TGF-β to establish a suppressive TME, ultimately reducing the sensitivity of T cells to ICIs (105, 106).

Sorafenib and lenvatinib, as commonly used multi-kinase inhibitors in HCC (107, 108), their treatment process is closely related to the intestinal flora. For example, in triple therapy (local treatment combined with lenvatinib and PD-1 inhibitors), the intestinal flora has been identified as a potential biomarker and affects treatment response (109). Meanwhile, the intratumoral flora shows a significant decrease in microbial diversity compared with adjacent non-tumor hepatic tissues, which may further regulate the immune microenvironment via the gut-liver axis and modulate treatment sensitivity to lenvatinib-PD-1 inhibitor combinations (110, 111), thereby affecting treatment outcomes such as progression-free survival (PFS) and overall survival (OS) in advanced HCC patients (112).

## 6 Regulatory effects of gut microbiota metabolites on HCC immunity

### 6.1 Immunomodulatory effects of SCFAs (functional classification and concentration dependence)

SCFAs—primarily acetate, propionate, and butyrate—are major fermentation products of dietary fiber by the gut microbiota. They regulate the immune balance in HCC via G protein-coupled receptors (GPR41, GPR43) and epigenetic mechanisms (113). Their effects are bidirectional and context-dependent, varying with disease status and metabolite concentration.

In terms of immune activation, butyrate is the most extensively studied SCFA. By inhibiting HDAC activity, butyrate upregulates the expression of MHC-I on tumor cells, thereby enhancing antigen presentation. In HCC models, butyrate also induces the secretion of CXCL11, recruiting *CD8* ^*+*^ T cells via the CXCL11–CXCR3 axis, and increases the production of IFN-γ and granzyme B by these T cells, strengthening their cytotoxicity (114–117). Propionate activates GPR43 signaling in DCs, promoting DC maturation and the secretion of IL-12, which further drives Th1 immune responses (118). However, SCFAs can also exert immunosuppressive effects. In non-alcoholic steatohepatitis (NASH)-related HCC, high concentrations of acetate promote Treg proliferation via the GPR43–STAT3 pathway, while Treg-derived IL-10 suppresses the cytotoxicity of *CD8* ^*+*^ T cells (119). Clinically, fecal acetate levels in NASH–HCC patients correlate positively with the proportion of Tregs, suggesting that the immune effects of SCFAs should be evaluated in the context of disease type and metabolite concentration (120).

### 6.2 Immune regulatory network of bile acid metabolites

Secondary bile acids—including DCA, lithocholic acid (LCA), and their derivatives—are generated by the microbial metabolism of primary bile acids and regulate HCC immunity via FXR, VDR, and TGR5 signaling (121, 122). Their effects exhibit structural specificity and are tightly linked to the composition of the gut microbiota, forming a “microbiota–bile acid–immunity” regulatory network.

Pro-immunity bile acids include ursodeoxycholic acid (UDCA), which promotes the polarization of M1 macrophages through TGR5 signaling, increasing the production of IL-12 and TNF-α while inhibiting the infiltration of M2 macrophages (123). In HCC patients, UDCA treatment increases the infiltration of *CD8* ^*+*^ T cells in the TME, a change that correlates with prolonged PFS (124). Chenodeoxycholic acid (CDCA) upregulates the expression of CXCL16 in liver sinusoidal endothelial cells through FXR activation, thereby recruiting CXCR6^+^ NKT cells and enhancing innate immune surveillance (105).

Conversely, some secondary bile acids exhibit immunosuppressive properties. DCA impairs the antigen-presenting capacity of DCs by downregulating the expression of CD80/CD86 (125). The LCA derivative isoalloLCA promotes Treg differentiation through the epigenetic activation of *Foxp3*, and its abundance correlates with the accumulation of PD-1^+^ T cells in HBV-related HCC (126).

The composition of the gut microbiota critically influences bile acid profiles. Enzymes such as bile salt hydrolase (BSH) and hydroxysteroid dehydrogenase (HSDH)—produced by bacteria of the genera *Clostridium* and *Bacteroides*—determine the synthesis of secondary bile acids. For example, *Akkermansia muciniphila* reduces DCA levels and the infiltration of M2 macrophages (127–129), whereas the overgrowth of *Escherichia coli* increases DCA accumulation, enhancing immunosuppression (130).

### 6.3 Immune effects of endotoxins and related metabolites

LPS—a key component of the cell walls of Gram-negative bacteria—together with other pro-inflammatory metabolites, mediates the progression of HCC driven by the gut microbiota. Their effects are dose- and time-dependent, balancing short-term immune activation against long-term immune tolerance (131, 132). At low concentrations (10–50 ng/mL), LPS activates Kupffer cells via the TLR4–MyD88 pathway, inducing the secretion of IL-6 and transiently promoting adaptive immune responses (133, 134). However, chronic exposure to high LPS concentrations (>100 ng/mL) induces immune tolerance, characterized by the upregulation of PD-L1 and IL-10, which inhibits the proliferation of *CD8* ^*+*^ T cells (135, 136). In HCC patients, LPS levels in the portal vein correlate positively with the accumulation of MDSCs in the liver, serving as an independent risk factor for poor prognosis (137, 138).

Other metabolites also contribute to immune dysregulation. Branched-chain amino acid metabolites such as isovaleric acid—produced by *Ruminococcus*—stimulate cancer-associated fibroblasts (CAFs) to secrete IL-8 via NF-κB, promoting the recruitment of neutrophils and their pro-tumor polarization (139, 140). Extracellular polysaccharides from *Enterococcus faecalis* inhibit complement activation, reducing the immunogenicity of tumor cells and facilitating immune evasion (141).

### 6.4 Immunomodulatory effects of indoles and derivatives

Indoles and their derivatives (e.g., indole-3-aldehyde, indole-3-propionic acid) are produced by gut microbes such as *E. coli* and *Limosilactobacillus reuteri* through the metabolism of tryptophan. Acting via AhR, these metabolites regulate immunity in HCC and represent potential targets for immunotherapy (142).

Indole-3-aldehyde (I3A) exerts anti-evasion effects: it promotes the infiltration of *CD8* ^*+*^ T cells through AhR–CXCR6 signaling and reduces the expression of PD-1 (143). In HCC models, the transplantation of *L. reuteri* increases intratumoral I3A levels, and when combined with anti–PD-1 therapy, reduces tumor volume by more than 50% (41).

Indole-3-propionic acid (IPA) exhibits immune-protective effects (144). It enhances the cross-antigen presentation capacity of DCs, promotes the generation of memory *CD8* ^*+*^ T cells, and prolongs immune responses (145). Clinically, serum IPA levels in HCC patients correlate positively with responses to immunotherapy, suggesting its potential as a predictive biomarker (146).

## 7 Gut microbiota-targeted HCC immunotherapy strategies and clinical translation

Based on the critical role of the gut microbiota and its metabolites in regulating immunity in HCC, microbiota-targeted interventions represent a promising strategy to overcome therapeutic bottlenecks and improve patient prognosis (Table 2). Such interventions include FMT, probiotic supplementation, antibiotic/phage therapy, and dietary or prebiotic modulation. Together, these approaches offer multiple pathways to optimize the efficacy of immunotherapy.

**TABLE 2:** Summary of gut microbiota-based intervention strategies for HCC.

Category	Intervention strategy	Model	Core mechanism	References
Probiotics	Prohep® multi-strain mixture (2025)	MASLD-HCC mice	Activates AMPK, inhibits mTOR, and synergizes with sorafenib to reduce tumor number/volume	(180)
Probiotics	*Weizmannia coagulans* MZY531 (2025)	H22 HCC mice	Inhibits inflammatory cytokines, activates the AMPK-mTOR autophagy pathway, and reduces tumor weight by 46%–53%	(181)
Probiotics	*Lactobacillus acidophilus* (2024)	NAFLD-HCC mice	Secretes valeric acid to block the Rho-GTPase pathway, prevents the progression of NAFLD to HCC, and enhances the intestinal barrier	(182)
Engineered bacteria	PD-1@EcM (acid-responsive *E. coli*, 2025)	Orthotopic/metastatic HCC mice	Produces anti-PD-1 antibodies intratumorally, promotes CD8^+^ T cell infiltration, and achieves >70% tumor inhibition	(183)
Engineered bacteria	ASEc@PNPs (lysis-circuit *E. coli*, 2024)	HepG2 xenograft mice	Releases doxorubicin and antigens upon lysis, triggers IFN-γ and TNF-α-mediated immune responses, and accelerates tumor regression	(184)
FMT	Healthy donor FMT (2025)	Human flora-associated metastatic HCC mice	Restores *Anaerotruncus*, inhibits neutrophil NETs-mediated inflammation, and reduces metastatic nodules	(185)
Microbiome-derived Compounds	Purified valeric acid (2024)	NAFLD-HCC mice	Mimics the effect of *Lactobacillus acidophilus*, inhibits Rho-GTPase via GPR41/43, and reduces tumor burden by 60%–70%	(182)
FMT	NCT05750030 (“FAB-HCC”) (2025)	Humans	Single FMT administration prior to standard therapy (atezolizumab + bevacizumab) to sensitize tumors	(186)
FMT	FMT reverses drug Resistance (2025)	Humans	Sequential FMT to overcome HCC resistance to standard systemic therapy	(187)

FMT from immunotherapy responders to non-responders has been shown to increase the infiltration of *CD8* ^*+*^ T cells in tumors and partially restore ICI efficacy (147, 148). Preclinical models confirm that FMT enhances the activity of antigen-presenting cells via the cGAS–STING pathway, reversing T cell exhaustion (149). Probiotic therapy also demonstrates therapeutic potential. For instance, the oral administration of *A. muciniphila* improves the efficacy of anti–PD-1 therapy in non-responders (150); supplementation with *Bifidobacterium* enhances the infiltration of *CD8* ^*+*^ T cells and the inhibition of tumor growth when combined with ICIs (123, 151). However, strain specificity is critical—some studies report the enrichment of *Bifidobacterium* in non-responders, highlighting the need for precise strain selection. In addition, the selective use of narrow-spectrum antibiotics to eliminate harmful bacteria (e.g., *K. pneumoniae*), high-fiber diets to promote the growth of SCFA-producing bacteria, and prebiotics (e.g., inulin) to enhance the proliferation of beneficial bacteria may improve microbial community structure and therapeutic responses (152–155). Nonetheless, challenges such as protocol standardization, patient stratification, and long-term safety remain unresolved (156).

### 7.1 Application of FMT in HCC immunotherapy

FMT restores microbial balance by transferring fecal material from healthy individuals or immunotherapy responders to patients, reshaping the HCC immune microenvironment through multiple mechanisms: ① Activating the cGAS–STING pathway to promote DC maturation and the secretion of type I interferons, thereby enhancing antigen presentation (157, 158); ② Reducing the intratumoral infiltration of MDSCs and Tregs, along with the levels of the inhibitory cytokines IL-10 and TGF-β (32, 159); ③ Repairing the integrity of the intestinal barrier, thereby reducing the translocation of LPS to the liver and inflammation-driven immune evasion.

However, FMT faces significant limitations: the lack of standardized donor screening (e.g., whether HLA matching is necessary), the potential transmission of pathogens (e.g., drug-resistant bacteria, viruses), and variable patient responses. Future research should integrate metagenomic sequencing for precise donor selection and develop standardized protocols to ensure the stability and safety of FMT (145).

### 7.2 Probiotic intervention strategies

Probiotics modulate the intestinal ecosystem through colonization, altering microbial composition and secreting immunomodulatory metabolites. Their advantages include safety and ease of standardization, but their efficacy is highly strain-specific, necessitating precise selection. (1) Akkermansia muciniphila enhances crosstalk between DCs and T cells by upregulating the production of IL-12, and when combined with anti–PD-1 therapy, reduces tumor volume by 60% in HCC models (160). (2) Bifidobacterium promotes the secretion of IFN-γ by CD8^+^ T cells via isobutyric acid and correlates positively with responses to ICIs in HBV-related HCC (161). (3) Limosilactobacillus reuteri alleviates T cell exhaustion through the I3A–AhR pathway, with particularly strong effects in NASH-related HCC (162, 163).

In HCC patients with high *Bifidobacterium* abundance, isobutyric acid levels correlate with the activity of *CD8* ^*+*^ T cells, the secretion of IFN-γ, and the inhibition of JAK/STAT3 signaling, thereby suppressing tumor growth (164). Animal models confirm that *Bifidobacterium* or isobutyric acid synergizes with PD-1 blockade to reduce tumor volume. Nevertheless, caution is warranted: certain *Lactobacillus* strains may upregulate M2 markers (CD163, CD206, HMOX1) and anti-inflammatory cytokines while downregulating M1-related cytokines (IL-1β, IL-12, TNF-α) (165). Long-term use of such strains may also disrupt microbial balance, emphasizing the need for dynamic, personalized dosing strategies.

### 7.3 Antibiotic and phage therapy

Targeted elimination of pro-tumor microbes is another strategy to relieve immunosuppression. However, broad-spectrum antibiotics impair microbial diversity and reduce ICI efficacy (166, 167). Narrow-spectrum antibiotics such as rifaximin are recommended, ideally administered 1 week prior to ICI initiation, to selectively eliminate pro-tumor bacteria (e.g., Enterobacteriaceae) while minimizing disruption of immune activation (31).

Phage therapy offers more precise targeting of pathogenic microbes. CRISPR–Cas9 editing can be used to engineer phages for the selective elimination of pathogenic species such as *Ruminococcus*. For instance, CRISPR–Cas12a achieves higher gene-editing efficiency in phages than Cas9, enabling the deletion of non-essential fragments to optimize phage function (168). Anti-CRISPR proteins (e.g., AcrIIC4) can fine-tune CRISPR–Cas9 activity, further improving phage specificity (169, 170). Despite its promise, phage therapy remains in the preclinical stage, with major challenges including phage resistance, delivery efficiency, and tumor-specific targeting.

### 7.4 Dietary and prebiotic regulation

Diet profoundly influences the gut microbiota and immune metabolism, representing a cost-effective and accessible intervention. High-fiber diets increase SCFA production and improve immunotherapeutic outcomes. In a prospective cohort, HCC patients consuming more than 25 g/day of dietary fiber showed a median OS prolonged by 9.2 months after immunotherapy (28.6 vs. 19.4 months), with fecal butyrate levels correlating positively with the activity of *CD8* ^*+*^ T cells (171). However, in NASH-related HCC, excessive fermentable fiber may exacerbate bile acid accumulation, underscoring the need for personalized dietary adjustments (172).

Prebiotics such as inulin and galactooligosaccharides promote the growth of SCFA-producing bacteria (e.g., Lachnospiraceae). In HCC models, inulin supplementation increases intratumoral butyrate levels and inhibits tumor growth (173). Clinical data suggest that prebiotics improve ICI response rates and correlate positively with gut α-diversity, supporting their use in combined interventions.

Specific dietary components that can positively regulate the intestinal microbiota include fiber-rich foods such as whole grains, legumes, fruits, and vegetables. These foods can promote the production of SCFAs, which are metabolites generated by intestinal microorganisms through the fermentation of indigestible dietary fiber. SCFAs have strong anti-inflammatory properties and are key factors in maintaining the intestinal barrier and regulating the microbiota (174, 175). Additionally, fermented foods like yogurt and kimchi can introduce beneficial probiotics, serving as a dietary approach to regulate the composition and function of the intestinal microbiota (176). In contrast, diets high in saturated fats, refined sugars, and processed foods are associated with reduced microbial diversity and an increase in pro-inflammatory taxa. This is because high saturated fat intake can exacerbate intestinal dysbiosis, disrupt intestinal integrity, and trigger a pro-inflammatory state (177, 178). Moreover, reduced consumption of fermentable fiber can limit the number of SCFA-producing bacteria, leading to microbial imbalance and inflammation (179).

## 8 Summary

The present review systematically summarizes the mechanisms through which the gut microbiota regulates immune homeostasis in HCC via the gut–liver axis. HCC patients exhibit characteristic patterns of dysbiosis, including the enrichment of *Proteobacteria* and the depletion of butyrate-producing bacteria. By disrupting the intestinal barrier, promoting chronic inflammation, modulating the function of immune cells (e.g., *CD8* ^*+*^ T cells, macrophages, Tregs), and altering the profiles of metabolites (e.g., SCFAs, bile acids), the gut microbiota shapes the TME, drives immune evasion, and influences responses to immunotherapy. Furthermore, we discuss microbiota-targeted therapeutic strategies such as FMT and probiotic supplementation.

The gut microbiota also shows promise as a biomarker for HCC monitoring. For instance, in early-stage HCC, 13 genera (e.g., *Parabacteroides*, *Gemmiger*) are enriched, while 12 genera (e.g., *Alistipes*) are reduced (24). In advanced HCC, increased levels of *Enterococcus* and Enterobacteriaceae, accompanied by reduced *Actinobacteria* and *Bifidobacterium*, correlate with advanced disease staging and poor prognosis (188). In addition, the abundance of *Streptococcus* and *Shigella* progressively increases with HCC progression (189).

Nevertheless, HCC surveillance remains inadequate: fewer than one-quarter of at-risk individuals undergo regular monitoring. Current diagnostic approaches—such as ultrasound combined with alpha-fetoprotein detection—exhibit limited sensitivity for early HCC, particularly in patients with cirrhosis or obesity (190). In contrast, gut microbiota signatures may provide novel opportunities to improve the early detection and monitoring of HCC.

For patients with HCC, practical strategies to improve the quality of the gut microbiota include adopting a high-fiber and Mediterranean diet. This dietary intervention can serve as an effective means to regulate the gut microbiota and help manage the risk of HCC (191, 192); incorporating strain-specific probiotics and prebiotics under medical guidance, which, by evaluating the contribution of probiotics to immunotherapy, offer potential therapeutic options that are safe and cost-effective (193); avoiding unnecessary antibiotics to prevent the exacerbation of gut dysbiosis, which has been shown to promote HCC progression through inflammatory, metabolic alteration, and immune regulation pathways (194); and regularly monitoring the microbial composition through fecal metagenomic analysis using methods such as 16S rDNA sequencing and metabolomics. This enables early characterization and use as biomarkers, thereby supporting personalized microbiota-targeted interventions, which are achieved by summarizing drug resistance mechanisms and proposing customized strategies (111, 195).

## 9 Challenges

Current research on the gut microbiota in the context of HCC faces several challenges. First, the microbiota of individual patients is shaped by multiple factors and exhibits substantial interindividual variability, which limits the clinical translation of microbiota-based biomarkers. Moreover, features such as reduced microbial diversity and the expansion of facultative anaerobes are not unique to HCC (196), reducing the disease specificity of these biomarkers. Second, HCC surveillance and screening rates remain low, while existing diagnostic methods lack sufficient sensitivity—particularly for early-stage disease—thus failing to meet clinical needs.

Mechanistic studies also present limitations. Most studies are based on animal or *in vitro* models, with insufficient validation of microbiota–gene associations (e.g., *CD6*, *MAPK10*) in human cohorts. The dynamic interactions among the microbiota, immunity, and tumors remain incompletely understood. Technical bottlenecks also exist: metagenomic sequencing still lacks strain-level resolution, and microbial DNA contamination in single-cell spatial transcriptomics hinders precise mechanistic analysis. From a clinical perspective, integrating microbiota monitoring into the management of HCC is crucial. This includes regularly performing fecal metagenomic sequencing to assess changes in microbial diversity and pathogenicity, metabolomic analysis of SCFAs and bile acids, as well as correlating microbiota data with systemic inflammatory markers such as IL-6 and CRP, thereby better stratifying patients and formulating personalized treatment strategies.
